# Fibromyalgia in women: association of inflammatory plasma proteins, muscle blood flow, and metabolism with body mass index and pain characteristics

**DOI:** 10.1097/PR9.0000000000001042

**Published:** 2022-10-04

**Authors:** Bijar Ghafouri, Emelie Edman, Marie Löf, Eva Lund, Olof Dahlqvist Leinhard, Peter Lundberg, Mikael Fredrik Forsgren, Björn Gerdle, Huan-Ji Dong

**Affiliations:** a Department of Health, Medicine, and Caring Sciences, Pain and Rehabilitation Centre, Linköping University, Linköping, University Sweden; b Department of Health, Medicine, and Caring Sciences, Linköping University, Linköping, Sweden; c Department of Biosciences and Nutrition, Karolinska Institutet, Huddinge, Sweden; d Department of Radiation Physics, and Department of Health, Medicine, and Caring Sciences, Linköping University, Linköping, Sweden; e Center for Medical Image Science and Visualization (CMIV), Linköping University, Sweden; f AMRA Medical AB, and Department of Health, Medicine, and Caring Sciences, Linköping University, Linköping, Sweden

## Abstract

**Introduction:**

Obesity is a common comorbidity in fibromyalgia (FM). Both FM and obesity have been connected to low-grade inflammation, although it is possible that previously reported inflammatory alterations in FM primarily may be linked to increased body mass index (BMI).

**Objective:**

This study aimed to investigate whether the inflammatory plasma protein profile, muscle blood flow, and metabolism and pain characteristics (clinical parameters and patient-reported outcome measurements) differed between female patients with FM with and without obesity.

**Methods:**

Patients with FM underwent clinical examinations, physical tests, and answered questionnaires. They were dichotomized according to BMI (<30 kg/m^2^ [n = 14]; ≥30 kg/m^2^ [n = 13]). Blood samples were collected and analyzed using a panel of 71 inflammatory plasma proteins.

**Results:**

There were significant (*P* < 0.05) differences in blood pressure, pulse, max VO2, pain intensity, physical capacity, and Fibromyalgia Impact Questionnaire between the groups; the obese group had higher blood pressure, pulse, pain intensity, and Fibromyalgia Impact Questionnaire. There were 14 proteins that contributed to the group belonging. The 4 most important proteins for the group discrimination were MIP1β, MCP4, IL1RA, and IL6, which showed higher concentrations in obese patients with FM. Significantly decreased blood flow and increased concentration of pyruvate were detected in obese patients compared with nonobese patients. There was significant correlation between inflammatory proteins and sedentary behavior and health status in obese patients with FM.

**Conclusions:**

These findings suggest that metabolism and inflammation interact in female patients with FM with obesity and might cause chronic low-grade inflammation. Screening for obesity and monitoring of BMI changes should be considered in the treatment of patients with FM.

## Abstract 2

Supplemental Digital Content is Available in the Text.

Metabolism and inflammation interact in fibromyalgia with obesity that can lead to chronic low-grade inflammation.

## 1. Introduction

Fibromyalgia (FM) is a condition with chronic widespread musculoskeletal pain, generalized hyperalgesia, and allodynia.^2,50^ Fibromyalgia has a prevalence of 2% to 8% in the general population, and it is more common in women.^22,35,51^ Patients with FM often experience impaired cognitive functions, sleep disturbances, mild to pronounced fatigue, anxiety, and depression.^23,28^ The quality of life is significantly reduced, and their health care–related and societal costs are high.^7^ The etiology of FM is not yet determined, and the pathophysiology is presumed to be a complex interplay of peripheral and central sensitization,^5,8,38^ with a lowered nociceptive threshold.^41,47^ Neuroinflammation, which implies that immune activation can modulate the excitability of nociceptive pathways, and small fiber neuropathy have been reported to influence and cause peripheral sensitization.^42,54^ Low-grade inflammation and altered cytokine profiles have been reported in FM.^9,17^ Significantly altered levels of inflammatory plasma proteins and analgetic and anti-inflammatory endocannabinoid lipid mediators have been detected when comparing patients with chronic pain conditions with healthy controls, suggesting that the differences are related to FM.^19,43,52^ Altered metabolism and blood flow in muscles, potentially associated with mitochondrial dysfunction, have been found in FM.^20^ These findings together might reflect an expression of ongoing low-grade inflammation in patients with FM.

Obesity and overweight have a prevalence of 60 to 70% in patients with FM.^10,24,31^ Pain and obesity are multifactorial conditions with a reciprocal relationship where they seem to influence each other in a negative manner.^12,39^ Obesity might cause pain through mechanical loading and low-grade chronic inflammation because fat has endocrine functions and secretes proinflammatory cytokines such as IL-6 and TNF-α,^29^ which have been reported to be elevated in FM.^19^ Proinflammatory cytokines can give rise to a hyperalgesia state^24,29^ because they contribute to the generation of mediators such as prostaglandins, histamine, and bradykinin, which interacts with the nervous system to induce pain.^29^ Conversely, pain could contribute to obesity through a more sedentary lifestyle and pain-associated avoidance behavior as well as through hedonic eating, which leads to temporary analgesic effects and can also constitute a coping mechanism.^29^ Pain and obesity become part of a vicious circle, which deteriorate the patients' health,^12^ because both high body mass index (BMI) and FM have been associated with inflammation.^37,52^ Considering that high BMI up to obesity level (≥30 kg/m^2^) is prevalent in FM, it is important to elucidate whether the markers of inflammation found in previous studies on patients with FM are in fact related to BMI (partly). Therefore, this study investigates the inflammatory protein profile in patients with FM with and without obesity. The primary aim is to investigate whether there are differences in inflammatory plasma protein profile and pain characteristics. The secondary aim is to investigate correlations between inflammatory plasma proteins with pain characteristic parameters in patients with FM with obesity.

## 2. Materials and methods

### 2.1. Study design

This study is part of a larger project that was a case–control design, investigating peripheral biochemical alterations in blood, saliva, and muscle and the correlation with central nervous system alterations in patients with FM and healthy age- and sex-matched control subjects.^20,45^ In this study, only patients with FM are included.

### 2.2. Study population

A total of 33 female patients with FM were recruited from the Pain and Rehabilitation Centre at the University Hospital in Linköping, Sweden. The inclusion criteria were female, FM diagnosis in accordance with the American College of Rheumatology (ACR) criteria 1990,^50^ and age in the range of 20 to 65 years. The exclusion criteria were contraindication for magnetic resonance examination, pregnancy, metabolic disease, rheumatoid arthritis, cardiovascular disease, lung disease, neurological disease, malignancy, severe psychiatric condition, and harmful consumption of alcohol, which corresponds to a score ≥6 in the Alcohol Use Disorder Identification Test.^3^ The inability to follow instruction for the 48-hour medical washout, refrainment from caffeine and nicotine for 12 hours, before sampling were also requirements. The patients were also instructed to avoid any heavy exercise for 48 hours before sampling.

Six participants were excluded because they did not provide blood samples. The remaining subjects (n = 27) were classified according to the World Health Organization (WHO) criteria: 18.5 to 24.9 = normal weight; 25.0 to 29.9 = overweight; 30.0 to 34.9 = mild obesity; and ≥35 = severe obesity (28). For the analyses, because the total number of participants was 27, they were divided into 2 groups regarding their BMI. The first group (labelled nonobese group) consisted of subjects with BMI <30 kg/m^2^, thus including both normal weight and overweight patients with FM. The second group (labelled obese group) consisted of subjects with BMI ≥30 kg/m^2^, hence consisting of patients with FM with mild and severe obesity; see Table 1.

**Table 1:** Patient-reported outcome measures (PROMs) obtained from health questionnaire.

Variables	Nonobese patients with FM		Obese patients with FM		Statistics
	n	Mean ± SD or median (min–max)	n	Mean ± SD or median (min–max)	*P*
Age (y)*	14	38.57 ± 12.62	13	43.85 ± 8.74	0.22
FM duration (y)†	14	3 (2–23)	13	8 (2–23)	0.06
Global pain intensity 7 d (NRS)*	13	5.85 ± 1.34	13	7.54 ± 1.66	0.01‡
HADS-depression*	14	6.86 ± 3.59	13	5.92 ± 3.62	0.51
HADS-anxiety*	14	7 ± 3.46	13	8.92 ± 3.90	0.19
HADS total*	14	13.86 ± 6.68	13	14.85 ± 6.34	0.70
PCS rumination*	14	6.57 ± 3.32	13	6.92 ± 4.54	0.82
PCS magnification*	14	3.71 ± 2.49	13	3.62 ± 2.22	0.91
PCS helplessness*	14	11.29 ± 4.65	13	11.62 ± 5.32	0.87
PCS total*	14	21.57 ± 9.09	13	22.15 ± 11.47	0.88
ISI total*	14	12.64 ± 6.46	13	16.15 ± 4.93	0.13
Sedentary behavior (hours/d)*	13	6.24 ± 2.47	12	5.48 ± 2.14	0.42
Physical activity-walking(days/wk)†	14	7 (0–7)	13	7 (3–7)	0.44
Physical activity-moderately strenuous activity (days/wk)†	14	2 (0–7)	13	1 (0–6)	0.58
Physical activity-very strenuous activity(days/wk)†	14	0 (0–7)	13	0 (0–5)	1.00
Physical capacity 30 min*	13	7.46 ± 3.15	13	5.08 ± 2.66	<0.05‡
PDI total*	14	35.57 ± 6.73	13	38.15 ± 14.58	0.57
LiSat-11 physical health†	14	2 (1–5)	13	2 (1–4)	0.65
LiSat-11 mental health†	14	4 (1–5)	13	3 (1–6)	0.57
LiSat-11 total*	14	41.29 ± 8.77	13	39.23 ± 9.06	0.56
FIQ total*	14	54.10 ± 14.10	13	66.23 ± 13.87	0.03‡

‡ Statistical significance.

* Normally distributed variables analyzed using the independent-samples *t* test and presented as mean (±SD).

† Not normally distributed variables analyzed using the Mann–Whitney *U* test and presented as median (minimum–maximum).

FM, fibromyalgia; n, number of participants; NRS, numeric rating scale; HADS, Hospital Anxiety and Depression Scale; PCS, Pain Catastrophizing Scale; ISI, Insomnia Severity Index; PDI, Pain Disability Index; LiSat-11, Life Satisfaction Questionnaire; FIQ, Fibromyalgia Impact Questionnaire.

All participants received verbal and written information about the study. Informed written consent was obtained, and the study was performed in accordance with the Helsinki Declaration. The study was approved by the Linköping University Ethics Committee (Dnr: 2016/239–31).

### 2.3. Procedure

The data were collected during visits at the Pain and Rehabilitation Centre in 2017 to 2018. At the first visit, clinical examinations and physical tests were performed on all participants. Between the first and the second visit, the participants answered a health questionnaire at home, the content of which is described below. At the second visit, microdialysis samples from trapezius muscle and blood samples were collected. The term “pain characteristic parameters” refer to variables obtained from clinical examinations and physical tests (clinical parameters) and variables obtained from the health questionnaire.

### 2.4. Clinical examinations and physical tests

All participants went through clinical examinations and physical tests. Measurements of height (m), weight (kg), and systolic and diastolic blood pressure (mm Hg) were performed by a research nurse. The aerobic fitness test (Åstrand test), which measures MaxVO2, was performed.^55^ For a detailed description of all tests, see Supplementary Methods (available at http://links.lww.com/PR9/A175).

### 2.5. Questionnaires

All participants answered a health questionnaire covering demographic and background data as well as containing multiple validated questionnaires regarding the psychosocial health. The participants stated their global pain intensity based on the last 7 days on a numeric rating scale (NRS) from 0 to 10, where 0 equaled no pain and 10 equaled worst possible pain. Patients with FM stated the year they were diagnosed with FM, which was converted into the variable FM duration.

### 2.6. Physical activity and sedentary behavior

The questionnaire included information about physical activity and sedentary behavior. These questions were taken from the Swedish National Board of Health and Welfare, and the questions were answered in a so-called open mode.^38^ One question was about the number of hours and minutes of sedentary behavior on an average day based on the last 7 days. Questions about the frequency (number of days per week) of walking as well as of moderately strenuous and vigorous physical activity based on the last 7 days were included. They were only allowed to consider the physical activities that lasted for at least 10 minutes. The participants also stated their self-perceived estimated level of physical capacity that they believed they could maintain for at least 30 minutes (physical capacity 30 minutes), on a scale from 1 to 18, where 1 equaled sitting down and 18 equaled aerobic exercise on elite level for women.

### 2.7. Sample collection

#### 2.7.1. Blood samples

Venous blood samples were collected in two 8-mL EDTA tubes. The samples were centrifuged at 1000*g* for 15 minutes, and the separate layers of plasma from the 2 blood samples, approximately 5 to 6 mL in total, were collected into a 12-mL Falcon tube and mixed gently. The plasma aliquoted in small portions and stored in −86°C until analysis.

### 2.8. Microdialysis sampling

Microdialysis is a well-established sampling technique for small molecules that has contributed to increased knowledge of peripheral nociceptive and metabolic mechanisms in chronic pain.^4,25,36,40^ The technique is based on a thin catheter with a semipermeable membrane that mimic the function of capillary blood vessel. The catheter is inserted in the trapezius muscle and perfused with a physiological saline solution. The small molecules in the interstitium are collected continuously by diffusion across the membrane along a concentration gradient.^44^ Microdialysis was conducted on trapezius muscle for 220 minutes. Samples were collected every 20 minutes, and subjects rated their pain (0 = no pain; 10 = worst imaginable pain) before catheter insertion, immediately after and every 20 minutes. The first 120 minutes after catheter insertion (the trauma period) was considered the time for tissue to recover from any trauma-induced changes in the interstitial environment. After this period, a 20-minute baseline period (denoted 140 minutes) followed by a 20-minute period of standardized repetitive low-force exercise on a pegboard (denoted 160 minutes) was performed. The experiment ended with a recovery period of 60 minutes during which participants rested in the armchair. For a detailed description, see Supplementary Methods (available at http://links.lww.com/PR9/A175).

### 2.9. Biochemical analysis

A commercially available panel of 71 proinflammatory and anti-inflammatory proteins (cytokines, chemokines, and growth factors) (U-PLEX, Meso Scale Discovery, Maryland) was used for biochemical analyzes on plasma. For a detailed description, see Supplementary Methods (available at http://links.lww.com/PR9/A175). The names of proteins and their limit of detections are listed in Supplementary Table 1 (available at http://links.lww.com/PR9/A175). The analysis of the pyruvate in microdialysis samples was made according to the methods presented in our earlier articles.^25^

### 2.10. Statistical analyses

The statistical program IBM SPSS Statistics (version 27) was used for univariate and bivariate statistics, whereas the program SIMCA (version 17; Sartorius Stedim Biotech, Umeå, Sweden) was used for multivariate data analysis. *P*-value <0.05 was considered statistically significant.

### 2.11. Univariate and bivariate statistics

Descriptive statistics were presented as mean values ± 1SD (normally distributed variables) or as median (minimum–maximum) (non-normally distributed variables). The differences between the 2 groups of patients with FM regarding the pain characteristic parameters were investigated using either independent-samples *t* test or Mann–Whitney *U* test for normally and non-normally distributed variables, respectively. Proteins that were detected in ≥50% of samples in one of the 2 groups were included in the statistical analyzes. In this case, only 2 of the 71 proteins in the panel were excluded because of this requirement, namely IL1β and TSLP. The levels of the remaining 69 proinflammatory and anti-inflammatory proteins were compared between the groups using the Mann–Whitney *U* test.

### 2.12. Multivariate data analysis

Multivariate data analysis is an exploratory method for investigating patterns in data. Multivariate data analysis is frequently used when dealing with omics data, such as proteomics^19,52^ where the number of variables significantly exceeded the number of participants.^48^ Principal component analysis was performed to evaluate the homogeneity of the data and to identify outliers.^53,54^ Hotelling's T2, which is a multivariate generalization of a 95% confidence interval, was used to identify strong outliers, and DModX (distance to model X) was used to identify moderate outliers. Orthogonal partial least squares discriminant analysis (OPLS-DA) was used to determine whether the plasma protein levels and the pain characteristic parameters differed between nonobese and obese patients with FM. For a detailed description, see Supplementary 1 (available at http://links.lww.com/PR9/A175).

GraphPad Prism (GraphPad Software LLC, San Diego, version 9.1.0) was used for the construction of graphs for selected proteins, with the highest VIP scores in OPLS-DA. Patients with FM were divided into normal weight, overweight, and obese (*x*-axis), and the median (range) of the concentration of proteins was displayed on the *y*-axis.

## 3. Results

### 3.1. Differences between nonobese and obese patients with fibromyalgia

#### 3.1.1. Questionnaire

The participants were between 22 and 56 years of age. The variables from the health questionnaires that differed significantly between the 2 groups were global pain intensity for the last 7 days, FIQ and estimated physical capacity (physical capacity 30 minutes). There were no significant differences between the groups regarding age, FM duration, anxiety, depression, pain catastrophizing, sleep disturbance, physical activity and inactivity, pain disability, or life satisfaction (Table 1). Smoking is a potential confounding factor when working with proinflammatory biomarkers. However, this factor was not considered in the analyzes because smoking was such an infrequent habit in this study population (n = 5). There were 3 obese subjects and 1 nonobese subject who reported smoking every day, and 1 nonobese subject with FM reported smoking less often than every day.

### 3.2. Clinical examinations and physical tests

Comparison of the 2 groups of patients with FM through bivariate analyzes showed significant increase in systolic and diastolic blood pressures and pulse, whereas maximal oxygen uptake (E-max VO2max), effort level, 30-second chair stand test (30CST), and the variables of grip strength and PPT did not differ significantly between the groups (Table 2).

**Table 2:** Clinical parameters obtained from clinical examinations and physical tests.

Variables	Nonobese patients with FM		Obese patients with FM		Statistics
	n	Mean ± SD or median (min–max)	n	Mean ± SD or median (min–max)	*P*
BMI (kg/m^2^)*	14	24.48 ± 3.12	13	35 ± 3.34	<0.01†
Blood pressure systolic (mm Hg)‡	13	120 (100–140)	13	130 (110–155)	0.02†
Blood pressure diastolic (mm Hg)‡	13	80 (60–80)	13	80 (70–100)	0.04†
Pulse (bpm)*	13	84.69 ± 9.66	11	96.91 ± 11.24	0.01†
Effort level (Borg scale)‡	14	7 (6–13)	10	7 (6–15)	0.69
30CST (number of times)‡	10	13.5 (10–23)	10	13 (6–16)	0.82
Max VO_2_ (ml O_2_/kg†min)‡	13	2.1 (1.5–3.4)	12	1.9 (1.6–2.9)	0.60
Mean grip strength maximal (N)*	14	250.54 ± 70.97	12	229.59 ± 73.07	0.47
Mean grip strength average (N)*	14	171.44 ± 60.14	12	149.47 ± 57.17	0.35
Mean grip strength endurance (N)*	14	145.56 ± 53.31	12	128.66 ± 51.53	0.42
Mean PPT trapezius right (kPa)‡	14	108 (38.33–281)	13	75.67 (24.33–258.67)	0.24
Mean PPT trapezius left (kPa)*	14	102.55 ± 54.56	13	85.23 ± 74.26	0.49
Mean PPT trapezius (kPa)*	14	117.15 ± 60.85	13	96.01 ± 76.17	0.43

* Normally distributed variables analyzed using the independent-samples t test and presented as mean (±SD).

† Statistical significance.

‡ Not normally distributed variables analyzed using the Mann–Whitney *U* test and presented as median (minimum–maximum).

FM, fibromyalgia; n, number of participants; 30CST, 30-s chair stand test; E-Max VO2, maximal oxygen uptake; PPT, pressure pain threshold.

### 3.3. Inflammatory plasma proteins

Principal component analysis was performed before the OPLS-DA and OPLS analyzes to detect any outliers in the data. One participant was identified as potential outliers based on Hotelling's T2. Retrospective data quality control revealed no major discrepancies in data, except for higher levels of some proteins in comparison with the other participants. Altogether, the exclusion of this participant from the subsequent analyzes was not justified.

**Figure 1.**
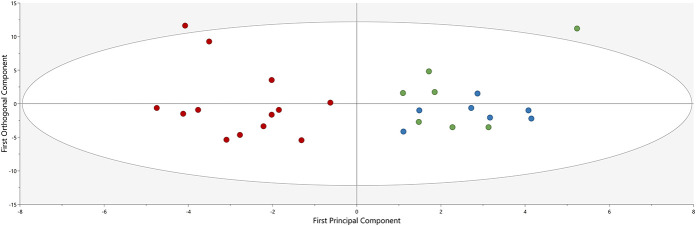
Score plot illustrating the discrimination analysis between obese (red circle) and nonobese (green and blue circle) patients.

A significant OPLS-DA model (1 predictive and 1 orthogonal component, *R*^2^ = 0.67, Q^2^ = 0.47, CV-ANOVA = 0.0058) containing 1 predictive component and 1 orthogonal component was obtained when comparing nonobese patients with FM with obese patients with FM (Fig. 1). The analysis was performed in 2 steps; in the first step, all variables were included in the analysis, and then in the second step, the variables with VIP ≥1.0 were used in a new OPLS regression. The score plot is colored for obesity (red circle), overweight (blue circle), and normal weight (green circle). The group with normal weight and overweight clustered together as a group of nonobese patients (Fig. 1). There were 14 proteins with VIPpred ≥1 and absolute *P*(corr) ≥0.3 that were important for group discrimination (Table 3). The proteins were positively associated with the obese group, and hence, all were upregulated in the group in obese patients compared with the nonobese group (ie, normal weight and overweight). To investigate the impact of smoking on inflammatory proteins, we reanalyzed the data excluding those study participants who reported smoking (3 obese and 2 nonobese). Differences remained significant (CV-ANOVA = 0.006, *R*^2^ = 0.74, Q^2^ = 0.55) between the obese and nonobese patients with FM. Only 1 protein GM-CSF (Table 3) showed VIP <1.

**Table 3:** Proteins important for group separation according to OPLS-DA sorted in descending order for VIPpred between obese and nonobese patients with fibromyalgia.

Variables	VIPpred	*P*(corr)	Nonobese patients with FM (n = 14)	Obese patients with FM (n = 13)	*P*
			Median (min–max)	Median (min–max)	
MIP1β	1.63	−0.67	26.41 (16.22–47.28)	43.96 (20.99–94.17)	0.01*
MCP4	1.53	−0.63	32.14 (24.56–58.80)	43.78 (31.27–95.14)	<0.01*
IL1RA	1.44	−0.59	112.74 (58.49–457.66)	277.06 (104.04–887.37)	<0.01*
IL6	1.39	−0.57	0.34 (0.12–1.16)	0.81 (0.44–2.20)	<0.01*
IP10	1.38	−0.57	122.46 (71.23–557.00)	221.23 (134.30–772.20)	0.01*
MIP1α	1.36	−0.56	12.85 (9.47–21.05)	18.20 (7.67–24.16)	0.03*
MCP1	1.24	−0.51	87.98 (64.90–162.63)	118.41 (49.21–244.27)	0.11
TARC	1.14	−0.47	21.76 (10.25–58.19)	29.14 (13.93–67.23)	0.24
IFNα2a	1.13	0.47	0.09 (0.00–36.12)	0.19 (0.00–0.66)	0.32
MCSF	1.11	−0.46	4.67 (2.35–10.79)	6.88 (3.00–14.10)	0.01*
TNFβ	1.09	0.45	0.12 (0.00–2.63)	0.13 (0.05–0.42)	0.92
MDC	1.09	−0.45	569.68 (231.59–918.04)	754.59 (486.41–1383.49)	0.01*
GMCSF	1.03	0.42	0.00 (0.00–0.55)	0.004 (0.00–0.14)	0.71
IL17C	1.01	−0.41	2.48 (0.87–4.98)	3.00 (0.26–12.31)	0.24

P-values marked with * refer to statistical significance.

### 3.4. Plasma protein levels in different classes of body mass index

**Figure 2.**
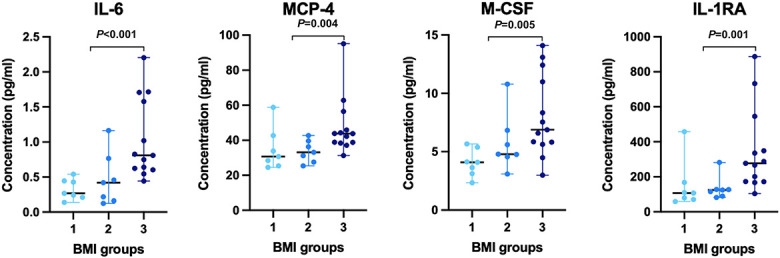
Plasma protein levels in different classes of BMI. *P*-values were obtained from the comparison between nonobese (ie, normal weight + overweight) and obese patients with FM through the Mann–Whitney *U* test. BMI group 1, group 2, and group 3 consist of subjects with normal weight, overweight, and obesity, respectively. The graphs present protein concentrations for the subjects as individual values and median with range. BMI, body mass index.

The levels of the 4 plasma proteins with the highest importance in the OPLS-DA, namely macrophage inflammatory protein 1-beta (MIP-1β), monocyte chemotactic protein 4 (MCP4), interleukin-1 receptor antagonist protein (IL1RA), and interleukin 6 (IL6) were compared between patients with normal weight (n = 7), overweight (n = 7), and obesity (n = 13) (Fig. 2). There was an increased protein levels with increased BMI for the selected proteins.

### 3.5. Plasma proteins and their correlation to sedentary behavior and impact

In the group with obese patients with FM, significant correlations (*P* < 0.05) were found between proteins and sedentary behavior and FIQ. The OPLS model (1 predictive component, R^2^ = 0.70, Q^2^ = 0.52, CV-ANOVA *P*-value = 0.037) of physical inactivity showed that 19 proteins (VIP > 1 and absolute *P*(corr) > 0.3) were multivariately correlated with sedentary behavior in obese patients with FM (Table 4). Of these 19 proteins, 17 were upregulated and 2 were downregulated in the group with obese patients with FM when compared with the group with nonobese (Table 4).

**Table 4:** Proteins correlated with sedentary behavior in obese patients with fibromyalgia (left columns).

Variables	VIP	*P*(corr)	Nonobese patients with FM (n = 14)	Obese patients with FM (n = 13)	*P*	Obese vs
			Median pg/mL (min–max)	Median pg/mL (min–max)		Nonobese
IL17D	1.672	0.525	6.784 (0–27.779)	7.022 (0–79.767)	0.808	↑
IL33	1.605	0.473	1.004 (0.548–2.308)	1.280 (0–4.279)	0.884	↑
MCP2	1.577	0.604	10.684 (7.800–44.228)	16.401 (8.340–29.866)	0.010*	↑
IL3	1.562	0.3077	10.499 (0–36.914)	15.768 (0–83.652)	0.242	↑
IL17B	1.561	0.303	1.154 (0.267–2.853)	1.173 (0.291–6.150)	0.467	↑
IL17F	1.557	0.439	1539.862 (774.797–2697.677)	2156.448 (250.921–5153.160)	0.409	↑
IL17EIL25	1.556	0.358	3.337 (1.835–10.661)	4.746 (0.449–14.321)	0.528	↑
IL31	1.524	0.3242	15.950 (10.017–26.537)	20.743 (0–41.786)	0.734	↑
MCP4	1.524	0.576	32.137 (24.560–58.795)	43.781 (31.277–95.143)	0.004*	↑
IP10	1.513	0.5769	122.464 (71.229–557.002)	221.230 (134.299–772.197)	0.007*	↑
IL22	1.494	0.338	1.412 (0.601–4.574)	1.860 (0.354–5.000)	0.771	↑
IL13	1.475	0.300	6.785 (2.720–13.359)	7.378 (4.254–13.713)	0.884	↑
TARC	1.474	0.750	21.766 (10.252–58.192)	29.145 (13.934–67.230)	0.244	↑
MCP1	1.415	0.438	87.978 (64.905–162.632)	118.412 (49.210–244.275)	0.109	↑
Eotaxin	1.381	0.348	60.504 (31.350–113.147)	63.455 (32.583–126.611)	0.438	↑
I309	1.345	0.582	12.938 (8.861–24.762)	14.058 (7.998–26.922)	0.264	↑
IL27	1.259	0.456	298.406 (174.367–706.290)	273.689 (128.797–633.226)	0.662	↓
MIP1β	1.075	0.440	26.415 (16.221–47.276)	43.958 (20.990–94.175)	0.012*	↑
IL29IFNL1	1.067	0.630	23.412 (2.683–52.888)	12.630 (2.937–26.235)	0.042*	↓

FM, fibromyalgia. In the right columns are presented the concentrations of the proteins in the 2 subgroups of patients with FM including the statistical comparisons between the 2 subgroups. P-values marked with * refer to statistical significance.

**Figure 3.**
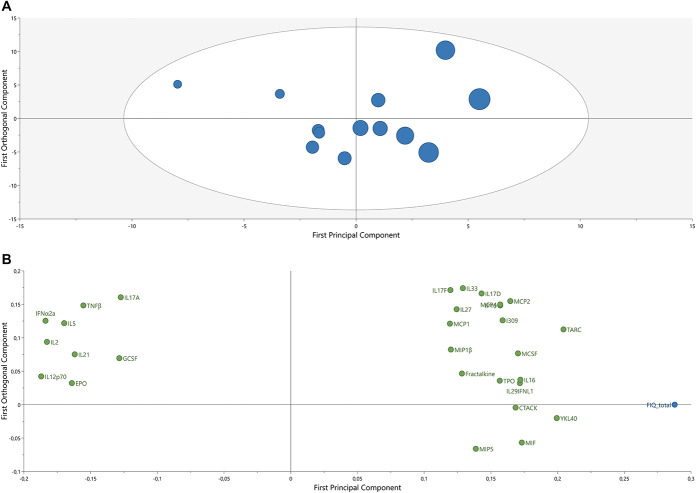
Fibromyalgia Impact Questionnaire (FIQ) and associated plasma proteins in obese patients with FM. In the score plot (left), the principal component (*x*-axis) shows the separation between the group of obese patients with FM based on FIQ, and the orthogonal component (*y*-axis) shows variation within the group. The circle size represents the FIQ scores of the subjects: The larger the circle, the higher the FIQ score. The color is based on BMI: blue = mild obesity; red = severe obesity. The tolerance ellipse is the equivalence of a 95% confidence interval and is based on Hotelling's T2. In the loading scatter plot (right), the variables are expressed as loadings, and only the variables with VIP ≥ 1 and absolute *P*(corr) ≥ 0.3 are presented. FM, fibromyalgia.

The OPLS model (1 predictive component and 1 orthogonal component, *R*^2^ = 0.90, Q^2^ = 0.66, and CV-ANOVA *P*-value = 0.047) showed that 29 proteins (VIPpred > 1.0; absolute *P*(corr) > 0.03) were multivariately correlated with an FIQ score in obese patients with FM (Table 5). The model displayed separation of the subjects with lower FIQ scores and the subjects with higher FIQ scores in the score scatter plot (Fig. 3). Nine proteins, namely IFNα2a, IL2, IL12p70, IL5, TNFβ, IL21, EPO, IL17A, and TNFα, were associated with lower FIQ scores. These 9 proteins were upregulated in the group with obese patients with FM when compared with the nonobese patients with FM. Twenty-four plasma proteins were associated with higher FIQ scores. Of these 24 proteins, 21 were upregulated and 3 were downregulated in the group with obese patients with FM when compared with the group with nonobese patients with FM.

**Table 5:** Proteins correlated with Fibromyalgia Impact Questionnaire in obese patients with fibromyalgia (left columns).

Variables	VIPpred	*P*(corr)	Nonobese patients with FM (n = 14)	Obese patients with FM (n = 13)	*P*	Obese vs
			Median (min–max)	Median (min–max)		Nonobese
IL12p70	1.621	−0.686	0.092 (0.006–0.765)	0.132 (0.039–0.312)	0.344	↑
IFNα2a	1.594	−0.675	0.091 (0.000–36.127)	0.188 (0–0.665)	0.319	↑
IL2	1.585	−0.671	0.090 (0.000–0.985)	0.104 (0–0.989)	0.496	↑
IL5	1.473	−0.623	0.192 (0.037–1.110)	0.202 (0.100–0.395)	0.903	↑
EPO	1.424	−0.603	52.785 (18.051–130.695)	62.445 (32.144–215.381)	0.120	↑
IL21	1.403	−0.594	89.154 (40.330–304.852)	105.925 (24.113–1106.754)	0.497	↑
TNFβ	1.347	−0.570	0.121 (0.000–2.637)	0.127 (0.052–0.424)	0.923	↑
GCSF	1.114	−0.472	7.347 (4.569–24.044)	8.818 (6.822–14.617)	0.076	↑
IL17A	1.105	−0.468	0.016 (0.000–16.557)	0.176 (0–7.200)	0.537	↑
TARC	1.772	0.750	21.766 (10.252–58.192)	29.145 (13.934–67.230)	0.244	↑
YKL40	1.73	0.732	17026.790 (15694.988–21810.430)	18356.758 (12091.898–19981.269)	0.081	↑
MIF	1.501	0.635	19466.546 (6795.524–30506.499)	25558.276 (8934.845–34167.281)	0.159	↑
IL16	1.491	0.631	106.601 (34.924–292.711)	143.787 (82.230–210.355)	0.073	↑
IL29IFNL1	1.489	0.630	23.412 (2.683–52.888)	12.630 (2.937–26.235)	0.042*	↓
MCSF	1.477	0.625	4.671 (2.349–10.789)	6.879 (2.996–14.098)	0.005*	↑
CTACK	1.461	0.618	808.720 (611.868–1900.329)	855.557 (525.954–1618.577)	1.000	↑
MCP2	1.426	0.604	10.684 (7.800–44.228)	16.401 (8.340–29.866)	0.010*	↑
I309	1.376	0.582	12.938 (8.861–24.762)	14.058 (7.998–26.922)	0.264	↑
IP10	1.362	0.576	122.464 (71.229–557.002)	221.230 (134.299–772.197)	0.007*	↑
MCP4	1.360	0.576	32.137 (24.560–58.795)	43.781 (31.277–95.143)	0.004*	↑
TPO	1.358	0.575	235.087 (103.596–412.361)	257.110 (64.964–431.847)	0.382	↑
IL17D	1.241	0.525	6.784 (0.000–27.779)	7.022 (0–79.767)	0.808	↑
IL33	1.117	0.473	1.004 (0.548–2.308)	1.280 (0–4.279)	0.884	↑
Fractalkine	1.113	0.4716	5970.100 (3148.637–8234.841)	5431.445 (2423.132–6954.101)	0.085	↓
IL27	1.0769	0.4560	298.406 (174.367–706.290)	273.689 (128.797–633.226)	0.662	↓
IL17F	1.037	0.439	1539.862 (774.797–2697.677)	2156.448 (250.921–5153.160)	0.409	↑
MCP1	1.035	0.438	87.978 (64.905–162.632)	118.412 (49.210–244.275)	0.109	↑
MIP1β	1.040	0.440	26.415 (16.221–47.276)	43.958 (20.990–94.175)	0.012*	↑

FM, fibromyalgia. In the right columns are presented the concentrations in the 2 subgroups of patients with FM including the statistical comparisons between the 2 subgroups. P-values marked with * refer to statistical significance.

### 3.6. Microdialysis

**Figure 4.**
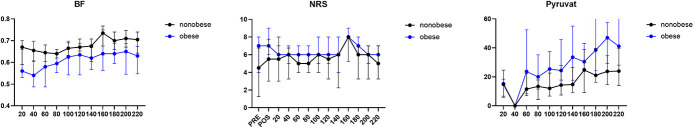
Results for blood flow (BF), pain intensity (NRS), and the metabolite pyruvate in trapezius muscle for obese patients (blue line) and nonobese patients (black line). Samples for BF and pyruvate from timepoints 20 to 120 minutes (trauma period), 140 minutes (baseline), 160 minutes (work), and 180 to 220 minutes (recovery period) were analyzed. NRS was rated even before catheter insertion (PRE) and immediately after catheter insertion (POST) and during the whole microdialysis experiment.

There were significant differences (*P* < 0.05) for mostly all timepoints in blood flow and pyruvate comparing obese and nonobese groups (Fig. 4). The obese patients rated higher pain intensity (NRS) during the whole microdialysis session. The time for recovery period after the work session with pegboard was longer for the obese patients compared with nonobese patients.

## 4. Discussion

There were 3 major results in this study. First, several pain characteristic parameters and inflammatory plasma proteins differed between obese and nonobese women with FM. Second, inflammatory plasma protein patterns correlated both with sedentary behavior and Fibromyalgia Impact Questionnaire (FIQ) in obese patients with FM. Third, the most important inflammatory plasma proteins for class discrimination showed increased concentrations with increased BMI.

There were significant (*P* < 0.05) differences in blood pressure, pulse, pain intensity, physical capacity, and FIQ between the groups; the obese group had higher blood pressure, pulse, pain intensity, and FIQ. The coexistence of these signs of metabolic syndrome in FM is in accordance with previously reported studies.^26^ However, there are limited numbers of studies, which reported that these signs of metabolic syndrome are associated with measured inflammatory protein biomarkers in blood. Results have been contradictory when investigating whether an association exists between BMI and pain characteristic parameters such as pain intensity, fatigue, and anxiety.^10,24^ Nevertheless, the study by Correa-Rodríguez et al. found that overweight and obese women with FM had significantly higher FIQ scores than those with normal weight and that BMI was significantly associated with FIQ score.^10^ Koçyiğit et al. reported significant differences in pain intensity, tender point count, level of depressive symptoms, and FIQ scores when comparing normal weight, overweight, and obese female patients with FM.^24^ Hence, it has been suggested that achieving normal BMI could be of importance in the management of clinical manifestations of FM (49).

There were 14 proteins that contributed to the group belonging. All these proteins were involved in cytokine activity (Gene Onthology: 0005125)^33^ and have been shown to be important as mediators of neuroinflammation associated with chronic pain.^53^

Four most important proteins for group discrimination were MIP1β, MCP4, IL1RA, and IL6, which showed higher concentrations in obese patients with FM. These proteins are involved in the inflammatory response. Because higher pain intensity previously has been connected to higher BMI,^12,24^ pain intensity can be viewed as a potential confounding factor. However, an OPLS model of pain intensity in the group with obese patients was not significant, suggesting that the significant inflammatory proteins in the OPLS-DA model are in fact connected to BMI and not majorly affected by pain intensity. When the FM group was divided into 3 BMI categories (normal weight, overweight, and obesity), a significant increase of the 4 cytokines was detected with increased BMI.

Based on the OPLS score plot for FIQ in the obese group, 2 subgroups could be identified; the participants with mild obesity were more cohesive compared with the participants with severe obesity, who seem to constitute a rather heterogeneous group with more scattered protein levels in comparison. As it is shown in Figure 2, protein concentrations for IL6, MCP4, MIP-1β, and IL1RA showed that the obese subgroup has a considerably wider range of protein concentrations for these 4 proteins than the nonobese subgroup. This observation suggests that patients with FM with higher BMI might constitute a heterogenous subgroup in regard of the protein concentrations.

In obese patients with FM, we found significant correlations between inflammatory proteins and sedentary behavior and health status. Associations between sedentary behavior and biomarkers of low-grade systemic inflammation have been reported for several chronic disease.^1,34^

Altered levels of metabolites and proteins in trapezius and vastus lateralis muscle in patients with FM compared with healthy subjects have been reported previously using microdialysis.^18,20,32^ Gerdle et al.^20^ reported significant muscle metabolic and blood flow alterations in this patient cohort compared with healthy controls, which may indicate muscle mitochondrial dysfunctions in FM. The present results show that the increased muscle metabolism and blood flow may be related to obesity because the concentration of pyruvate was significantly higher in obese patients with FM compared with nonobese patients with FM. Interestingly, we found that the recovery period for the muscle in patients with FM after the working period was longer as the patients reported more pain and concentration of pyruvate was high and not normalized after the recovery period. Insertion of the microdialysis catheter in muscle causes an acute nociception, which can lead to an inflammatory response. However, if such inflammatory response would act on nociceptors and sensitize those is not clear, and different muscle may react differently regarding their pain-evoking properties.^30^ Moreover, the obese FM subgroup reported no changes in pain intensity after the catheter insertion, whereas the nonobese FM subgroup reported higher pain intensity after catheter insertion. Such differences in nociceptor reaction between obese and nonobese patients with FM need to be confirmed in a larger study.

The increased pyruvate concentration found in the obese subgroup may support the upregulated cytokine findings in obese patients with FM. It is well established that pyruvate induces oxidative stressors such as reactive oxygen species (ROS), which in turn can activate production of inflammatory cytokines. There is evidence that supports ROS contribution to the regulation of vascular tone and inflammatory signaling in obesity.^16^ Our results show that metabolism and inflammation are interacting in FM with obesity that can lead to chronic low-grade inflammation.

The pathogenetic mechanisms in FM are not fully understood, which complicate diagnosis and treatment.^21^ Gaining insight into the underlying peripheral biological processes involved in the FM condition, by studying muscle metabolism and plasma protein profiles and scrutinizing the pain characteristic parameters, could lead to increased understanding of the activated molecular mechanisms in FM and possibly to development of target approaches.

The vicious cycle of pain-inactivity-weight gain-more pain related to obesity has been identified as a difficult challenge in pain rehabilitation. Interdisciplinary pain rehabilitation programs are based on a biopsychosocial model of chronic pain. However, obese patients may not benefit from interdisciplinary pain rehabilitation programs to the same extent as their normal weight peers, eg, excess weight per se could be a barrier in rehabilitation, eg, to increase physical activity.^11,12^ Nutrition intervention might be beneficial on pain patients with comorbid obesity^6,14,27^ because the change of inflammation profiles in the blood indicates the change of chronic pain (central sensitization).^13,15,27,46^

This study has some limitations. The sample size was relatively small, and there were an insufficient number of subjects for comparing the 3 BMI groups (BMI < 25, BMI 25–29.9, and BMI ≥ 30). Therefore, normal weight and overweight patients with FM were merged into 1 group for comparison with the obese group. Comparison of 3 groups could have provided more insight into the relationship between BMI and levels of plasma proteins. Although high homogeneity is sought, the inclusion and exclusion criteria affect the study's applicability and generalizability. For example, including only women limits the generalizability of the results, but it yields a more homogeneous cohort and reduces the influence of potential sex differences. Although newer versions of the ACR criteria were available before the study,^49^ at the timepoint for data collection in this study, the 1990 ACR criteria were best recognized at most clinics in Sweden, including the Pain and Rehabilitation Centre in Linköping. In this pilot study, we only found patients with FM with obesity reported longer FM duration compared with nonobese patients (*P* = 0.06, medium effect size r = 0.36). Further investigation in a larger sample size will have the possibility to explore the correlation between obesity and FM duration. Furthermore, the impact of smoking when investigating inflammatory proteins in plasma should be considered in future studies.

## 5. Conclusion

In summary, this study shows that the inflammatory plasma protein profile, self-reported pain characteristics (pain intensity, impact, and physical capacity), and blood pressure, pulse, and physical fitness differed between female patients with FM with and without obesity. Furthermore, levels of inflammatory plasma proteins were multivariate correlated to an FIQ and sedentary behavior in patients with FM with obesity. These findings show obesity as comorbid condition in FM that significantly cause activation of inflammatory network in female patients with FM.

## Disclosures

The authors have no conflicts of interest to declare.

## Appendix A. Supplemental digital content

Supplemental digital content associated with this article can be found online at http://links.lww.com/PR9/A175.

## Supplementary Material

SUPPLEMENTARY MATERIAL
