# Supplementary material for: Fibromyalgia in women: association of inflammatory plasma proteins, muscle blood flow, and metabolism with body mass index and pain characteristics
Source: Pain Rep. 2022 Oct 4;7(6):e1042. doi: 10.1097/PR9.0000000000001042 (PMC9534367; doi:10.1097/PR9.0000000000001042)
Supplement: SUPPLEMENTARY MATERIAL [file painreports-7-e1042-s001.pdf]

Supplementary Methods

Aerobic fitness test (Åstrands test)

To define the study participants maximal oxygen uptake (Max VO_2, ml O_2/kg*minute), they performed a submaximal cycle ergometer aerobic fitness test (Åstrands test) on a Monark 928 E cycle (Monark Exercise AB, Vansbro, Sweden).

Lower extremity strength

The lower extremity strength was assessed through the 30 second chair stand test (TST), which is a test that investigates the number of times a person can stand up from a sitting position in a standard chair under the time course of 30 seconds. Arms were crossed over the chest. In addition, the pulse (beats per minute) and saturation of oxygen (%) were measured, and the effort level estimated on the Borg scale, before (at resting state) the TST. The Borg scale is a 15-point scale used to estimate the perceived physical exertion, where 6 equals no exertion and 20 equals maximal exertion.

Hand strength

Grip strength was measured by using Grippit (AB Detektor, Gothenburg, Sweden), which is a device that can measure grip forces in Newton (N) during a 10 second trial. Grippit has sufficient reliability in female FM-patients. The maximal (peak) and average grip forces as well as the endurance-value at the end of the 10 second trial were registered for the dominant and the non-dominant hand. The mean values of the dominant and non-dominant hand are presented.

Pressure Pain Thresholds

Pressure pain thresholds (PPT) were measured with a manual pressure algometer (Somedic SenseLab AB, Södsdala, Sweden) that had a probe with a contact area of 1 cm^2. The pressure was applied on specific pre-marked points on the muscle belly of the examined muscles. The applied pressure was increased by 30 kPa/s until the maximum pressure of 600 kPa was reached (to avoid tissue damage) or the subject perceived the pressure as painful and pushed a stop-button. The muscles, which were examined bilaterally, were the *M. trapezius, M. erector spinae and M. tibialis anterior*. For each tested location, three trials were performed with the minimum interval of 30 seconds, and the PPT were defined as the mean value of these three trials. For analyzes in this study, we used the mean value for the right and left m. trapezius separately as well as the mean value for all measurements of m. trapezius bilaterally.

Psychological distress

Hospital Anxiety and Depression Scale (HADS) is an instrument to assess symptoms of anxiety (HADS-A) and depression (HADS-D). There are seven questions related to anxiety and seven questions related to depression. For each question there are four answer alternatives with the scores 0-3. Both HADS-A and HADS-D each generate a score between 0 and 21 points, which means that the total score ranges between 0 and 42 points. The higher the score the more likely the participant is to suffer from anxiety or depression, respectively.

Pain Catastrophizing Scale (PCS) is based on thirteen items rated on a five-point scale (0-4). Catastrophizing encompasses three dimensions which are all included in the PCS: rumination, magnification and helplessness. These three parts have the maximum scores of 16, 12 and 24, respectively, with a combined total score of 52 points.

Sleep disturbance

Insomnia Severity Index (ISI) is used to assess the degree of difficulty of the insomnia symptoms and how much impact they have on the affected person. It consists of seven items which are rated on a five-point scale (0-4). The maximal score of ISI is 28 points, and the higher the score the more severe are the symptoms.

Disability

Pain Disability Index (PDI) is used to estimate the degree of impact that long lasting pain has on activities in different important areas of life. The seven items that constitutes the PDI are work ability, family responsibilities, social activities, recreation, sex, personal daily activities, and basic life-sustaining activities such as breathing, eating, and sleeping. These seven items are rated on a ten-point numeric scale (1-10). The total score ranges between 7 and 70 points, where higher scores equal higher degree of disability.

Fibromyalgia Impact Questionnaire (FIQ) is used for the assessment of health status of women with FM. It consists of 10 items and assesses the impact of fibromyalgia on different activities, general wellbeing, and ability to work. FIQ also includes estimations of the level of pain, fatigue, stiffness, anxiety, and depression. Different rating scales are applied for different items within the questionnaire. When adding all the items there is a specific way to calculate the total score that ranges between 0 and 100 points, where higher scores indicate higher impact of fibromyalgia on the persons health.

Quality of life

The Life Satisfaction questionnaire (LiSat-11) encompasses the level of satisfaction a person experiences in different areas of life, for example life in general, personal economy, sexual life and physical and mental health. In total, the questionnaire consists of 11 items that are rated on a six-points scale (1-6), generating a total score between 11 and 66 points. A high score equals high satisfaction. We used the scores from the questions about physical health and mental health along with the total score of the questionnaire.

Microdialysis

Briefly, microdialysis catheter (20 kDa cut-off (CMA 60), membrane 30 mm length, 0.5 mm diameter, Microdialysis AB, Solna, Sweden) was inserted under ultrasonic supervision at half the distance between the seventh cervical spine and the lateral part of the acromion as described previously. The catheters were perfused with a syringe pump (CMA 107; CMA Microdialysis AB, Stockholm, Sweden) at 5 µl/minute with a solution resembling the muscle interstitial fluid (Ringer acetate solution; Fresenius Kabi AB, Uppsala, Sweden) containing 3 mM glucose, 0.5-mM lactate, and 3.0 µM [14C]-lactate (GE Healthcare, Buckinghamshire, UK). Furthermore, nutritive muscle blood flow was estimated by the MD ethanol technique using $3H_2O$ instead of ethanol[50].

Samples were collected every 20 min for 220 min. Immediately after the insertion of catheters, participants rested comfortably in an armchair for 120 min (i.e., the trauma period) to allow the tissue to recover from possible changes caused by catheter insertion. After the trauma period, participants continued to rest for 20 min, the baseline period (denoted 140 min). The baseline period was followed by a 20-min period of standardized repetitive low-force exercise of the neck- shoulder muscles sitting on a chair performed on a pegboard (denoted 160 min). The experiment ended with a recovery period of 60 min during which participants rested in the armchair. Subjects rated their pain intensity in trapezius muscle of the most painful side every 20 min using NRS.

Biochemical analysis

A commercially available panel of 71 pro- and anti-inflammatory proteins (cytokines, chemokines, and growth factors) (U-PLEX, Meso Scale Discovery, Maryland, USA) was used for biochemical analyzes on plasma. The multiplex immunoassay is based on MULTI-ARRAY technology which combines electrochemiluminescence detection and patterned arrays that is a classic sandwich-model where light emits upon electrochemical stimulation initiated at the electrode surface of the plate. The plasma samples were thawed and analyzed according to the manufacturer protocol. The antibody set contained specific antibodies for all

71 proteins in the panel. The antibody-samples were mixed with the linkers and 50 µl were pipetted to the 96-well (10 spots) plates, which the antibodies bound to and coated. The plates were incubated at 4 °C on a shaker with gentle shaking overnight. The unbound antibodies were washed away using 200 µl washing buffer (PBS, 0.05% Tween-20) three times. The plasma samples or the standard samples were added, and the plates were incubated for one hour at room temperature and then washed again. Detection antibodies conjugated electrochemiluminescent labels were added and the plate was incubated for 1 h. After the binding assay was completed, the plate was placed in the instrument where a voltage applied to the plate electrodes causes the captured labels to emit light. The chemiluminescence component allowed the detection of light intensity which was proportional to the amount of protein in the samples. The light intensity of all the different proteins examined was converted into concentrations (pg/ml) in a program using standard curves. To produce these standard curves, synthetic standard proteins in known concentrations were added in two rows of wells to measure their light intensity by chemiluminescence. Data were collected and analyzed using MESO QUICKPLEX SQ 120 instrument (Meso Scale Diagnostics LLC, Maryland, USA) equipped with DISCOVERY WORKBENCH data analysis software.

Multivariate data analysis

Orthogonal partial least squares (OPLS) analysis is a regression method that was applied to investigate correlation between specific pain characteristic parameters (y-variable) and the inflammatory plasma proteins (x-variables). The OPLS analyzes were performed in the group with BMI ≥ 30 since the purpose was to investigate the existence of correlations between inflammatory proteins and pain characteristic parameters in FM-patients with obesity.
In accordance with the guideline suggestion by Wheelock and Wheelock, the number of components, R^2 and Q^2 values and CV-ANOVA p-value for the different statistical models from the OPLS-DA and OPLS analysis are reported. These parameters are used for the evaluation of the model quality. R^2 estimates goodness of fit and Q^2 estimates goodness of prediction for the model, thus how well the model explains the data in the original data set and how well the model is expected to predict another data set with different subjects. R^2 is always higher than Q^2 but ideally, the difference between R^2 and Q^2 should preferably not exceed 0.3 according to the rule of thumb, since a larger difference implies overfitting of the model. Cross-validated analysis of variance (CV-ANOVA) was performed to determine the model significance.

Variable Influence on Projection (VIP) scores were used to determine which variables were important for the group separation. The higher the VIP-score, the more important is the variable for group separation, and VIP≥1 is commonly considered significant. When the model contained more than one component, the VIP predictive (VIP pred) was used. P(corr) was used as a complement to VIP and represent the loadings scaled into correlation coefficients for each variable, with the range -1 to +1. The closer the p(corr) is to -1 or +1, the stronger is the correlation. Variables with VIP > 1.0 and absolute p(corr) ≥ 0.3 are considered as significant variables in this study.

Supplementary Tables

Supplementary Table 1. The panel consisting of 71 inflammatory plasma proteins used in the study. *Proteins that were excluded since they were not detected in >50% of the samples in one of the two groups of FM patients. The protein synonyms are in parentheses. LLOD – ULOD = lower limit of detection – upper limit of detection.

Protein ID	Protein name	LLOD – ULOD (pg/ml)
Eotaxin	Eotaxin	3.2 – 4,800
Eotaxin3	Eotaxin 3	7.3 – 21,400
IL17AF	Interleukin-17AF	1.84 – 18,400
IP10 (CXCL10)	Interferon gamma-induced protein 10	0.49 – 6,000
MCP1	Monocyte chemotactic protein 1	0.74 – 6,600
MCP4	Monocyte chemotactic protein 4	7.5 – 3,800
MDC (CCL22)	Macrophage-derived chemokine	8.4 – 20,100
MIP1α (CCL3)	Macrophage inflammatory protein 1-alpha	7.7 – 4,200
MIP1β (CCL4)	Macrophage inflammatory protein 1-beta	1.5 – 1,600
TARC	Thymus and activation-regulated chemokine	0.51 – 2,200
EPO	Erythropoietin	1.8 – 20,000
FLT3L	Fms-related tyrosine kinase 3 ligand	0.49 – 6,000
IFNβ	Interferon-beta	3.1 – 100,000
IL17B	Interleukin-17B	0.79 – 4,000
IL17C	Interleukin-17C	2.2 – 20,000
IL17D	Interleukin-17D	4.8 – 40,000
IL1RA (IL1RN)	Interleukin-1 receptor antagonist protein	1.7 – 5,000
IL2Ra	Interleukin-2 receptor subunit alpha	10 – 55,000
IL3	Interleukin-3	11 – 16,000
IL9	Interleukin-9	0.14 – 1,500
IL17EIL25 (IL25)	Interleukin-17E/Interleukin-25	0.58 – 9,200
IL17F	Interleukin-17F	155 – 112,000
IL21	Interleukin-21	1.2 – 12,600
IL22	Interleukin-22	0.13 – 3,400
IL23 (IL23A)	Interleukin-23	1.4 – 21,600
IL27	Interleukin-27	9.6 – 50,600
IL29IFNL1 (IFNL1)	Interleukin-29/Interferon lambda-1	1.2 – 11,800
IL31	Interleukin-31	7.3 – 11,060
IL33	Interleukin-33	0.59 – 10,300
CTACK	Cutaneous T-cell-attracting chemokine	1.8 – 4,200
ENA78	Epithelial-derived neutrophil-activating protein 78	0.53 – 3,900
Fractalkine	Fractalkine	102 – 180,800
ITAC	Interferon-inducible T-cell alpha chemoattractant	1.5 – 5,100

IL17A	Interleukin-17A	2.6 – 23,400
MIP3α	Macrophage inflammatory protein 3 alpha	1.8 – 20,800
MIP3β	Macrophage inflammatory protein 3 beta	0.67 – 2,000
SDF1α	Stromal cell-derived factor 1 alpha	278 – 103,200
TNFα (TNF)	Tumor necrosis factor-alpha	0.54 – 3,700
VEGFA	Vascular endothelial growth factor A	2.0 – 4,900
GMCSF	Granulocyte-macrophage colony-stimulating factor	0.12 – 9,400
IFNγ	Interferon-gamma	1.7 – 17,000
IL10	Interleukin-10	0.14 – 3,700
IL12p70	Interleukin-12 p70	0.69 – 5,300
IL2	Interleukin-2	0.70 – 1,900
IL4	Interleukin-4	0.08 – 2,100
IL5	Interleukin-5	0.24 – 4,000
IL6	Interleukin-6	0.33 – 2,000
IL8	Interleukin-8	0.15 – 2,200
GCSF	Granulocyte colony-stimulating factor	1.6 – 20,400
IFNα2a (IFNA2)	Interferon-alpha-2a	4.0 – 42,400
IL12IL23p40 (IL12B)	Interleukin-12/Interleukin-23 p40	2.8 – 21,000
IL15	Interleukin-15	0.82 – 3,000
IL16	Interleukin-16	6.6 – 21,500
IL18	Interleukin-18	2.5 – 42,000
IL1α	Interleukin-1 alpha	0.98 – 5,100
IL7	Interleukin-7	1.5 – 7,000
TNFβ (LTA)	Tumor necrosis factor-beta	0.47 – 4,300
TPO	Thrombopoietin	19 – 40,400
Eotaxin2	Eotaxin 2	3.1 – 6,000
GROα	Growth-regulated alpha protein	0.25 – 2,500
I309 (CCL1)	T lymphocyte-secreted protein I309	6.8 – 3,000
IL13	Interleukin-13	3.1 – 1,900
MCSF	Macrophage colony-stimulating factor 1	0.29 – 2,000
MCP2	Monocyte chemotactic protein 2	0.11 – 2,000
MCP3	Monocyte chemotactic protein 3	0.79 – 5,000
TRAIL	TNF-related apoptosis-inducing ligand	0.66 – 10,000
MIF	Macrophage migration inhibitory factor	4.3 – 27,000
MIP5	Macrophage inflammatory protein 5	0.34 – 30,000
YKL40 (CHI3L1)	Chitinase-3-like protein 1	0.39 – 5,000
IL1β*	Interleukin-1 beta	0.15 – 3,800
TSLP*	Thymic stromal lymphopoietin	0.2 – 10,100
